# CellSim: a novel software to calculate cell similarity and identify their co-regulation networks

**DOI:** 10.1186/s12859-019-2699-3

**Published:** 2019-03-04

**Authors:** Leijie Li, Dongxue Che, Xiaodan Wang, Peng Zhang, Siddiq Ur Rahman, Jianbang Zhao, Jiantao Yu, Shiheng Tao, Hui Lu, Mingzhi Liao

**Affiliations:** 1 College of Life Sciences, Northwest A&F University, Yangling, Shaanxi China; 2 College of Information Engineering, Northwest A&F University, Yangling, Shaanxi China; 3 Department of Bioinformatics and Biostatistics, SJTU Yale Joint Center Biostatistics, Shanghai Jiao Tong University, Shanghai, China

**Keywords:** Cell similarity, Regulation network, Cell type identification, Cell heterogeneity, Human cancer cells

## Abstract

**Background:**

Cell direct reprogramming technology has been rapidly developed with its low risk of tumor risk and avoidance of ethical issues caused by stem cells, but it is still limited to specific cell types. Direct reprogramming from an original cell to target cell type needs the cell similarity and cell specific regulatory network. The position and function of cells in vivo, can provide some hints about the cell similarity. However, it still needs further clarification based on molecular level studies.

**Result:**

CellSim is therefore developed to offer a solution for cell similarity calculation and a tool of bioinformatics for researchers. CellSim is a novel tool for the similarity calculation of different cells based on cell ontology and molecular networks in over 2000 different human cell types and presents sharing regulation networks of part cells. CellSim can also calculate cell types by entering a list of genes, including more than 250 human normal tissue specific cell types and 130 cancer cell types. The results are shown in both tables and spider charts which can be preserved easily and freely.

**Conclusion:**

CellSim aims to provide a computational strategy for cell similarity and the identification of distinct cell types. Stable CellSim releases (Windows, Linux, and Mac OS/X) are available at: www.cellsim.nwsuaflmz.com, and source code is available at: https://github.com/lileijie1992/CellSim/.

## Background

Cell type and tissue specificity are key aspects of precision medicine and regenerative medicine researches [1].The cells direct reprogramming and complex human disease studies, such as cancer, show that cell-cell interaction networks and cell-specific regulatory differences are essentialfor researchers [2, 3].Direct reprogramming requires cellular similarity between original cell and the target cell type, as well as sharing regulation networks [4–6]. Cells similarity can be estimated by the position and function of the cell in vivo, but is infeasible for all human cell types and still highly challenging. Besides, due to the social pressures and sampling difficulties in part of human tissues and cell-types, direct assay of the cell and tissue-specific regulation networks is highly challenging [7]. Thus, the direct reprogramming cell types are limited [8]. Therefore, precise calculation of human cell types similarity and intracellular regulation networks will be of great help to the development of cell reprogramming techniques and complex disease treatment [9].

Traditional “wet” lab methods(molecular or cell experiments) can not meet the requirements for calculating the similarity of all human cell types since thousands of cell types have been confirmed in the human body [10].For instance, Cell Ontology provides a relationship between cells which contain a large number of cells among many species [11, 12]. BioGRID and HPRD database offer regulation networks in species [13, 14]. These data represent cells connection and global pathway function but cannot quantize cells relationship and distinguish the cell-specific regulation [15]. Bioinformatics methods are needed in similarity calculation. Successful methods, Mogrify [16],CellNet [17],MNDR [18], RAID [19] and ViRBase [20] can predict reprogramming factors and assess the fidelity of cellular engineering. There are also some other related soft or database for computational biology [21, 22]. However, these predictions are limited by the cell type numbers and cannot precisely calculate the similarity among all human cell types. Further, none of these resources can predict cell types by its specific expression genes and transcription factors (TFs).

eIn this study, we developed CellSim software in order to compute the cell similarity based on Cell ontology network and cell-specific regulation network in FANTOM [10, 23, 24]. We used the term in Cell Ontology as a node in cell network, and the relationship between each term as an edge. Moreover, CellSim acquires cell similarity based on the cell network with semantic similarity as a measurement to compute the similarity between each pair of nodes. Additionally, CellSim provides the detail TF-gene regulation relationships which are shared among original cell and the target cell. Considering the importance of cancer research and tumor heterogeneity which show specific molecular regulation mechanism and gene expression, CellSim divides the cell type-specific regulatory network into cancer and normal cell network respectively, in order to provide a more precise reference for cancer researches.

## Implementation

**Fig. 1:**
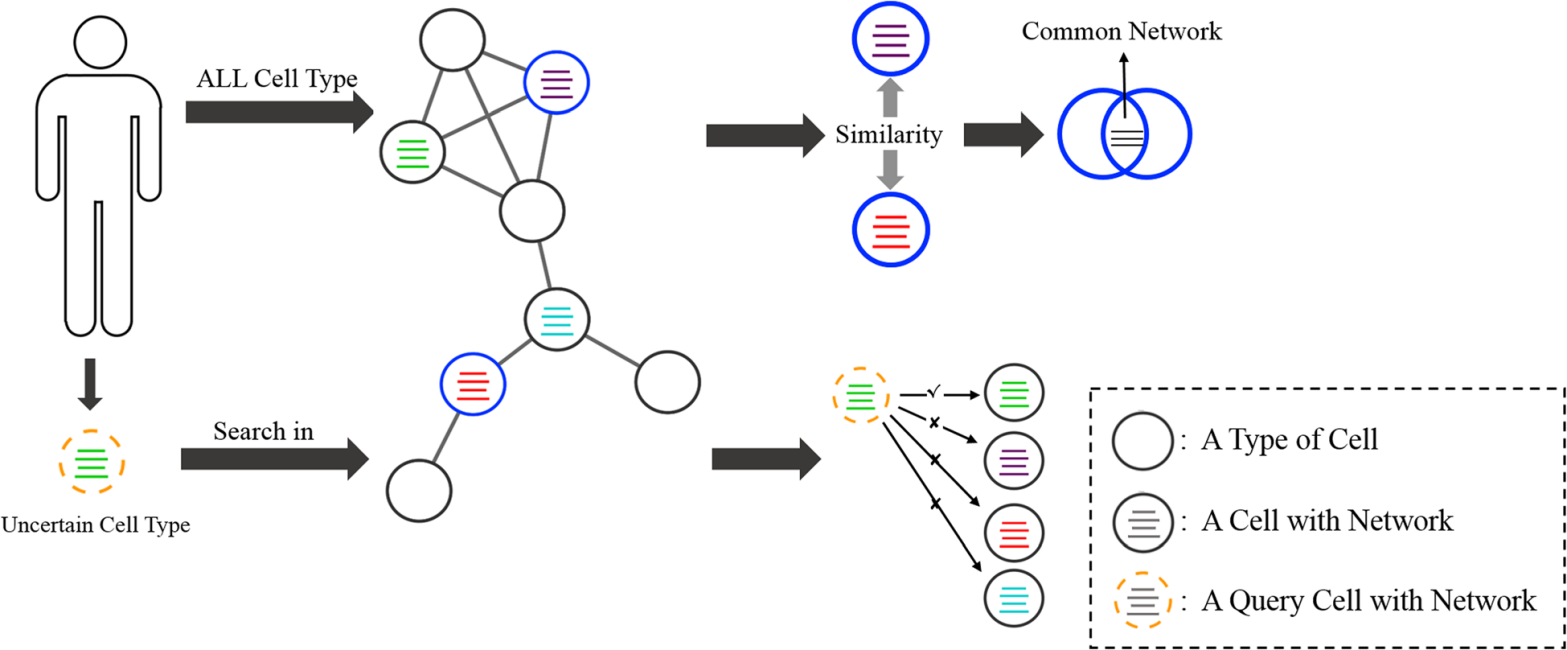
Schematic Diagram of CellSim. CellSim has two main functions:the first one is the calculation of cell similarity and the second one is the prediction of cell type

This version of CellSim was developed using the PYQT5 platform. The main workflow of CellSim is shown in Fig. 1. We extracted all human cell types from existing database, calculated similarities between cells, and integrated human tissue-specific TF-genes regulation networks to adjust and rectify similarity scores. CellSim can mainly achieve two functions. First, quantify the similarity between any human cells and provide part cells’ shared regulation networks which are sorted by the regulation reliability from high to low. Seconds predict cell types by cell-specific highly expressed genes in query cell and sort cells through the expected score. Considering the complexity of tumor cells, the prediction is performed in human healthy cells and tumor cells, separately.

### Cell similarity calculation

The networks of cell types were downloaded and analyzed from Cell Ontology which includes 2160 cell types(Including both general and branch cell types). The similarity score between different cells was calculated by semantic similarity algorithm [25–28], with formula as below:

1 $$ IC(t)=-\mathit{\log}P(t) $$

2 $$ {IC}_{ma}\left(t,{t}^{\prime}\right)=\underset{\widehat{t}\in Pa\left(t,{t}^{\prime}\right)}{\mathit{\max}} IC\left(\widehat{t}\right) $$

3 $$ sim\left(t,{t}^{\prime}\right)=\frac{2\ast {IC}_{ma}\left(t,{t}^{\prime}\right)}{IC(t)+ IC\left({t}^{\prime}\right)} $$

Where t refers to a cell type which is as a term in Cell Ontology. IC(t) refers to information content value of cell type t. P(t) refers to the percent that t and its progeny cell types are divided by all cell types. Pa(t, t^′^) refers to the cell types that contain both t and t^′^. IC_ma_(t, t^′^) refers to the maximum information content of paternal cell type node shared by t and t^′^.As the above definition, the scale of similar score is from 0 to 1.

**Fig. 2:**
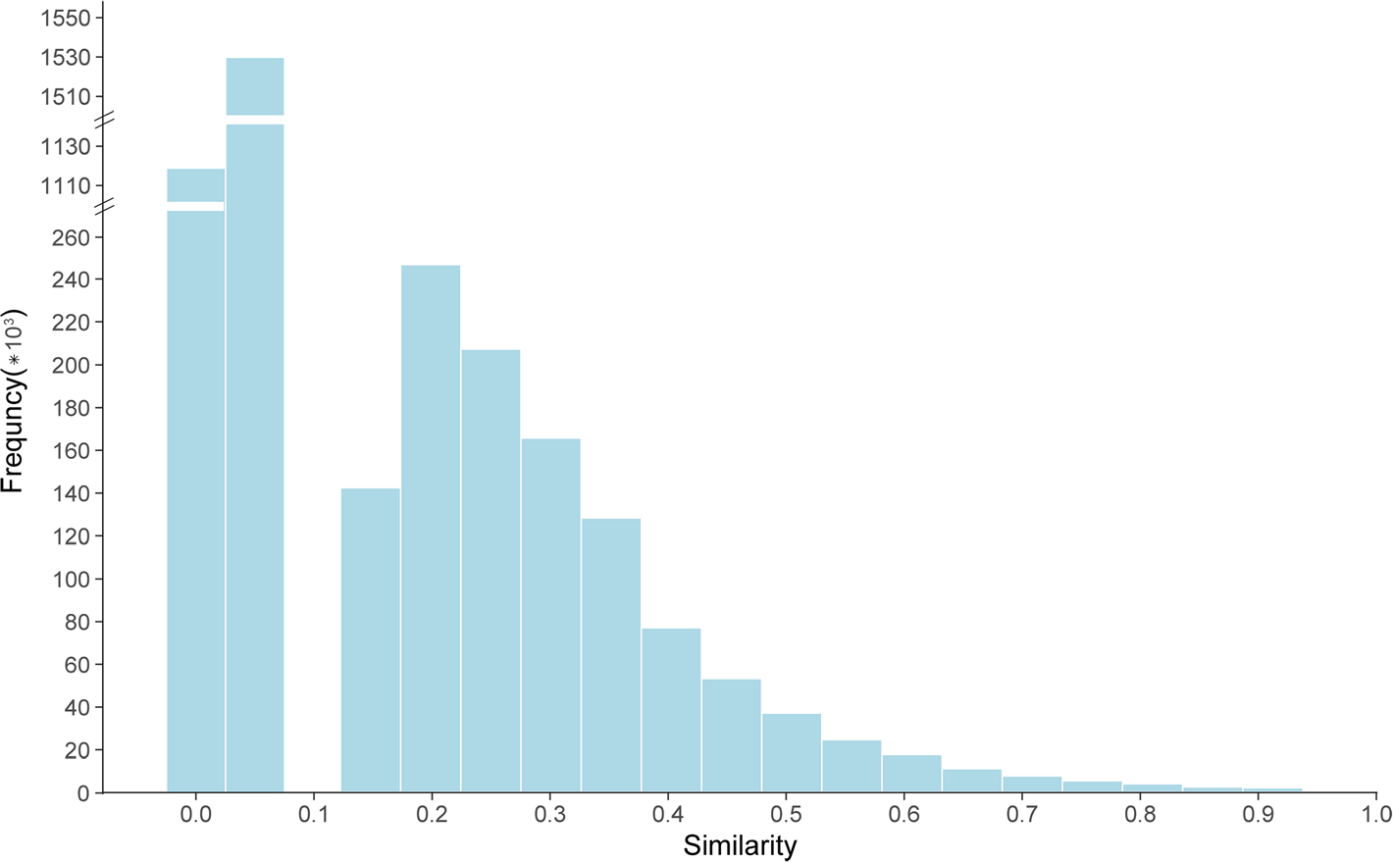
The distribution map of all human cell types similarity scores

**Fig. 3:**
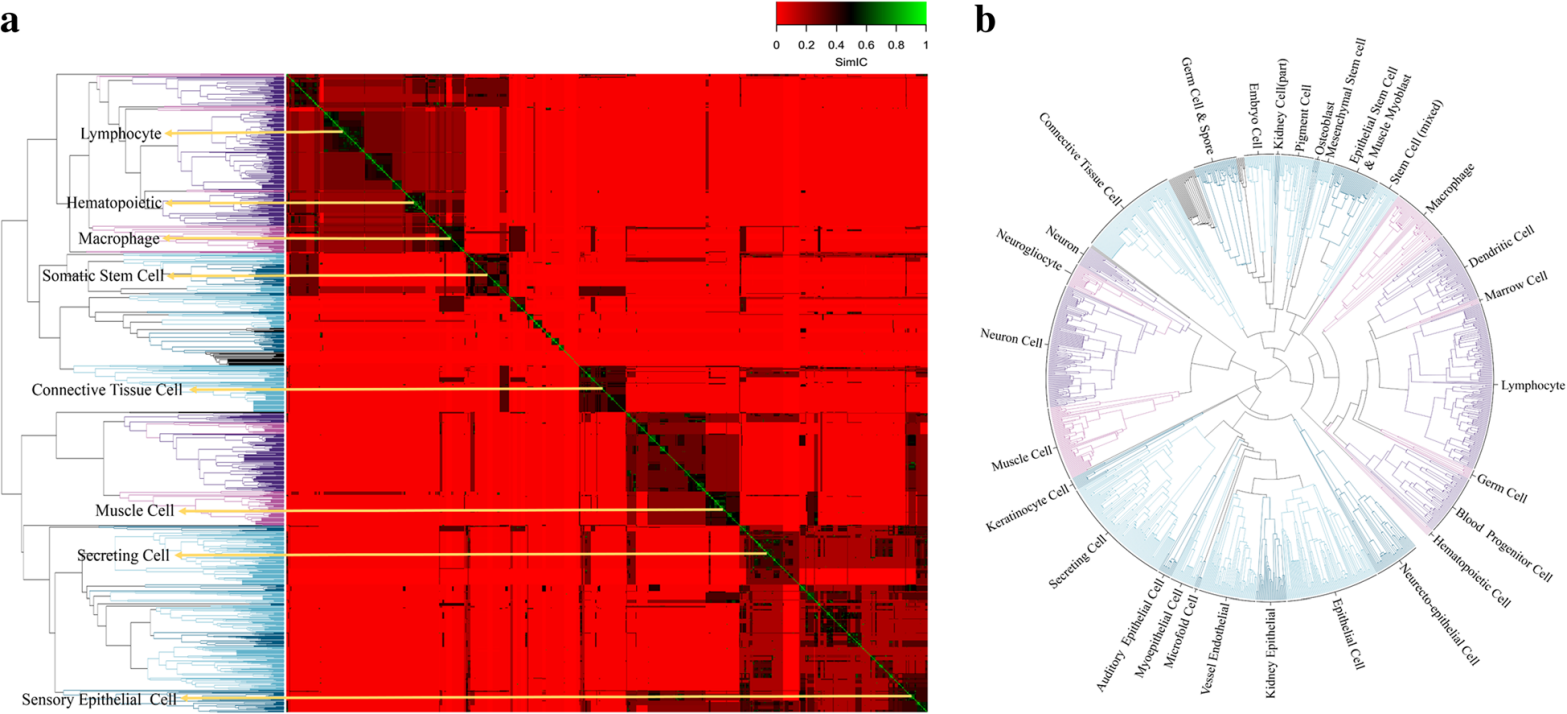
Human cell similarity cluster. **a** Human cell similarity heat map. The similarities of all human cell types were calculated by Lin’s semantic similarity arithmetic. Yellow lines were used to point out the modules with a high similarity. **b** Circles Hierarchical Clustering Diagramof Human cell Similarity. The clustered branches were annotated with alternated blue and cell names

We calculated the distribution of similarity scores across all cell types. The distribution of scores is given in Fig. 2. The distribution indicates that when the similarity scores are less than 0.1, the relationship between cells is weak and strangeness. Similarity is moderate when scores are between 0.1 and 0.4. Cells show a significant similarity when score is between 0.4–0.7. When the similarity score is higher than 0.7, it is considered that there is a strong correlation between the cells, which indicate there potential property, location and functional similarity or even belong to the same type of cells. Further more, we used Euclidean Distance [29] to cluster the cells with their similarity score. Results, including heat map and circle cluster figure, both of these are showing tidy phenomenon with apparent modules (Fig. 3), which indicates the reliable and accurate measure ability of our methods.

### Prediction of cell types with TF-gene regulatory network

**Fig. 4:**
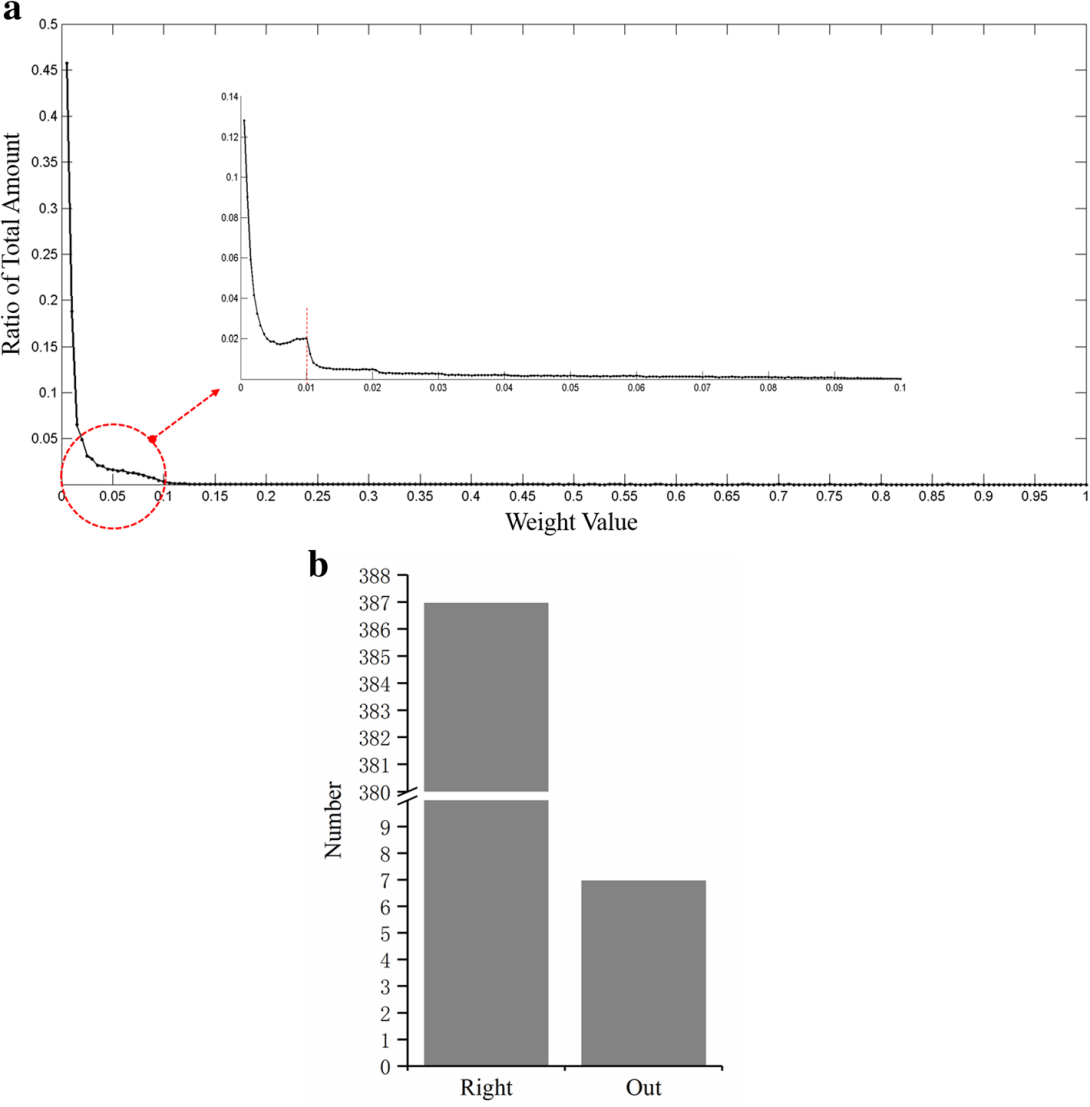
Cell-specific Network Filtration. **a** Confidence scores distribution diagram of cell-specific network in FANTOM. Results show that more than 98% diagrams reach a plateau at 0.01, which was then used as a threshold to get robust network. **b** The bar of cell networks with plateau at 0.01

**Fig. 5:**
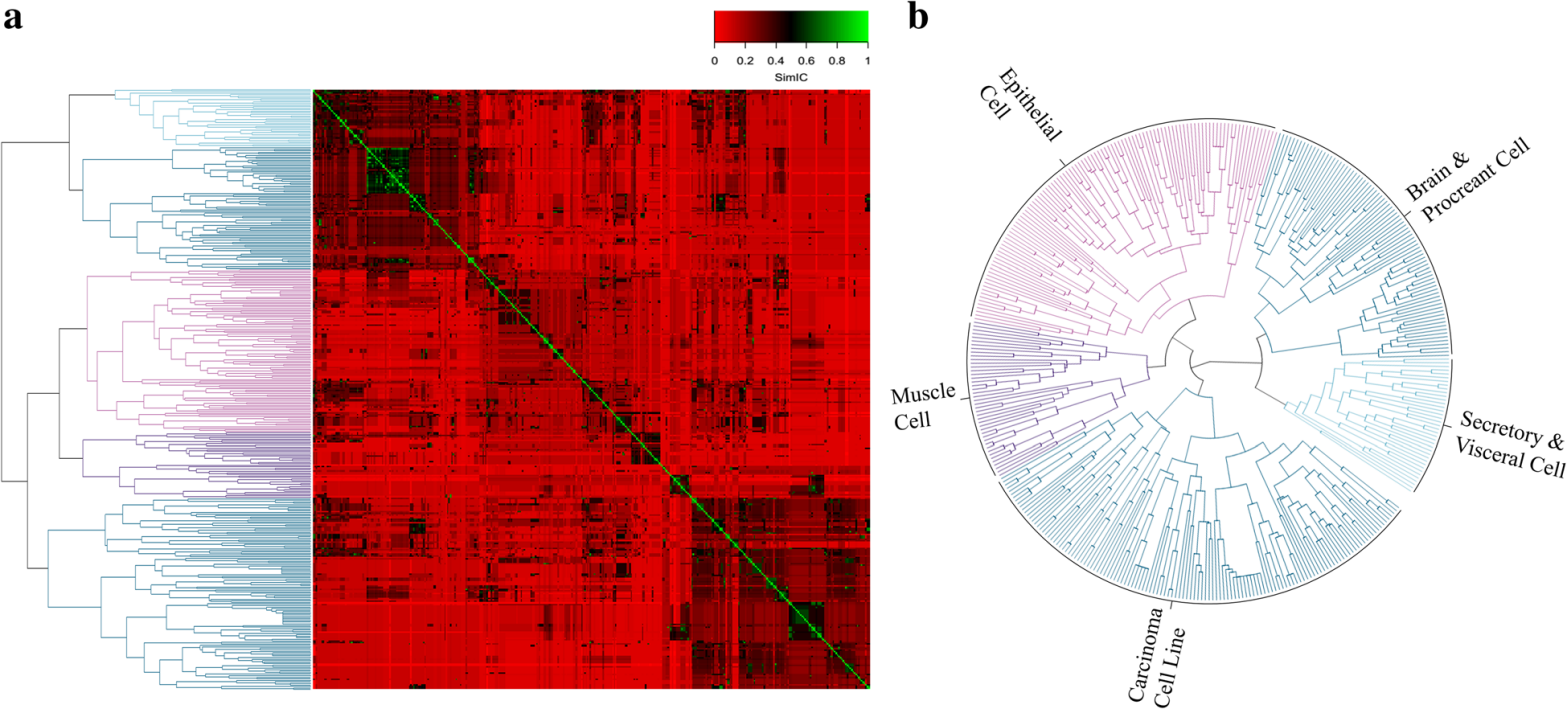
Cluster of Cells with Specific Network in FANTOM. The similarity of Cells with Specific Network in Fantom5 was calculated by Lin’s semantic similarity arithmetic. Then the cells were clustered and showed as heat map and hierarchical clustering diagram. (**a**) Heat map of clustered cells. (**b**) Hierarchical clustering diagram

We continued to validate our methods based on the cell-specific TF-gene regulatory networks in FANTOM project, which includes both 258 human normal cells and 130 cancer cells. As shown in the distribution of regulation reliability scores (Fig. 4a), there is an apparent fault at 0.01. We conjecture that the bellow regulations are weak or noise. And the statistic result shows that only 7 cells, less than 2%, do not follow the rule (Fig. 4b). Therefore, we removed the edges of which score was lower than 0.01 in order to get robust molecular networks. Finally, unique TF-gene edges were extracted as a cell-specific network for each type of cells. Our heatmap and circle cluster results also show high tidiness (Fig. 5). Based on the cell-specific networks, CellSim provides the prediction of cell types with a query gene list.

### Function design

CellSim provides two kinds of search entries, including cell types and gene list. For the first entry, when users input two records of cell types, CellSim will calculate and display the similarities between these two lists. If user inputs only one cell type, CellSim will calculate and show the similarity between this cell type and all the other types of cells. Besides, based on the cell-specific TF-gene regulation networks in FANTOM, CellSim can also provide the common network between different cells if there are the corresponding regulation networks in FANTOM. Another entry is a list of genes, through which function CellSim can predicate the gene related specific cell type. We used cell-specific TF-gene networks mentioned above as background datasets. CellSim provides both radar charts and the associated tables as results, which can be downloaded freely. *Net Map Radar Chart* is drawn according to the first row of the table, which represents the ratio of query genes and cell-specific genes to cell-specific genes (Formulas 4). *Gene List Map Radar Chart* is drawn according to the second row of the table, which represents the ratio of query genes and cell-specific genes to query genes (Formulas 5). The formulas are given bellow:

4 $$ R=\frac{Q\cap M}{num(M)} $$

5 $$ R=\frac{Q\cap M}{num(Q)} $$

Where R represents overlap scores between the query gene list and the specific genes in target cell type. Q represents the query gene list. M represents gene list of the cell-specific network. Num(M) means the number of genes in M.

## Result

### Stem cell similarity calculation as case study

**Fig. 6:**
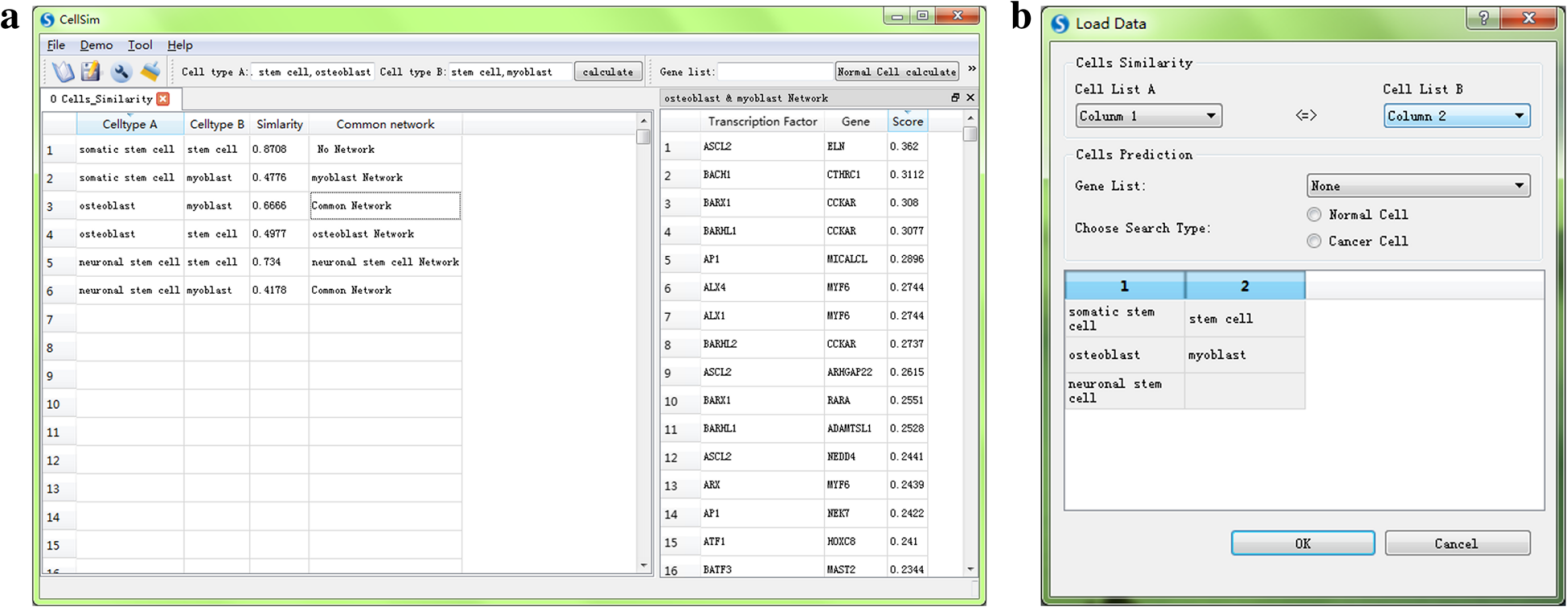
Example of cell similarity calculation. (**a**) The result tab in CellSim main interface. (**b**) File input window

We used somatic stem cell, stem cell, neuronal stem cell osteoblast, and myoblast as an example to show the similarity calculation results of cell types (Fig. 6). As shown in the figure, cell type can be inputted by file(Fig. 6b), or quickly entered in the primary interface. The results are presented on the primary interface of CellSim in the form of tabs (Fig. 6a). Precise data are shown in Table 1. The conventional network of cell types is annotated in the last column. If the two cell types have a shared network, it is filled in “Common Network”. If only one cell has a network, it is shown as the cell type’s name. Clicking the block in CellSim, the detailed information of the regulation network will be shown in a floating window and sort according to the regulation reliability scores. Specific regulation network sample is shown in Table 2.

**Table 1:** Cell types similarity and common networks

Celltype A	Celltype B	Similarity	Common network
somatic stem cell	stem cell	0.8708	No Network
somatic stem cell	myoblast	0.4776	myoblast Network
osteoblast	myoblast	0.6666	Common Network
osteoblast	stem cell	0.4977	osteoblast Network
neuronal stem cell	stem cell	0.734	neuronal stem cell Network
neuronal stem cell	myoblast	0.4178	Common Network

**Table 2:** The top ten regulation terms in sharing network of osteoblast and myoblast

Transcription Factor	Gene	Score
ASCL2	ELN	0.362
BACH1	CTHRC1	0.3112
BARX1	CCKAR	0.308
BARHL1	CCKAR	0.3077
AP1	MICALCL	0.2896
ALX4	MYF6	0.2744
ALX1	MYF6	0.2744
BARHL2	CCKAR	0.2737
ASCL2	ARHGAP22	0.2615
BARX1	RARA	0.2551
BARHL1	ADAMTSL1	0.2528
ASCL2	NEDD4	0.2441
ARX	MYF6	0.2439
AP1	NEK7	0.2422
ATF1	HOXC8	0.241
BATF3	MAST2	0.2344
ATF1	HOXC9	0.2203
ASCL2	TAS1R1	0.2198
BACH1	ADAMTSL1	0.2184

**Fig. 7:**
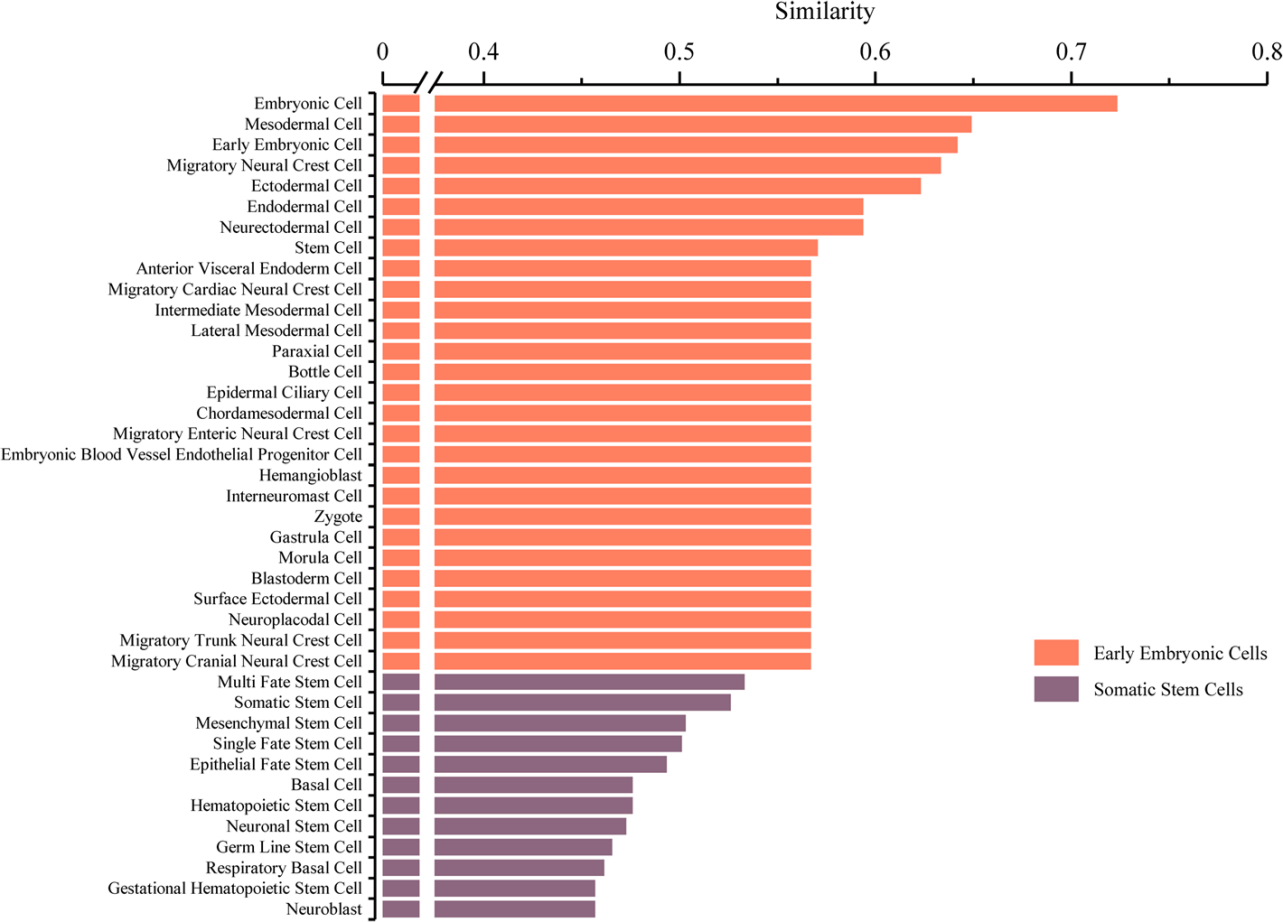
Embryonic stem cell similar cell types analysis

We analyzed the similar trend of embryonic stem cells (ESC) and extracted the top-ten similarity score cell types are shown in Fig. 7. The most similar to ESC is embryonic cell, mesodermal cell, and early embryonic cell, which have an identical feature to ESC, high pluripotency. This result also validates the reliability of CellSim. Besides, ESC is similar to migratory neural crest cell, neuroectodermal cell, migratory cranial neural crest cell, and migratory trunk neural crest cell. The similarity is lower than early embryonic cells and higher than normal somatic stem cells, which shows that ESC is more likely to differentiate into specific neural stem cells than other somatic stem cells. The results indicate that the most similar cell types are early embryonic cells and followed by adult stem cells, which is consistent with the pluripotency difference instem cell types [30, 31]. This consequence proves the reliability and robustness of CellSim. We speculate that ESCs and related neural stem cells have similar regulation networks and functions, which needs further experimental validation.

### Cell type prediction

**Fig. 8:**
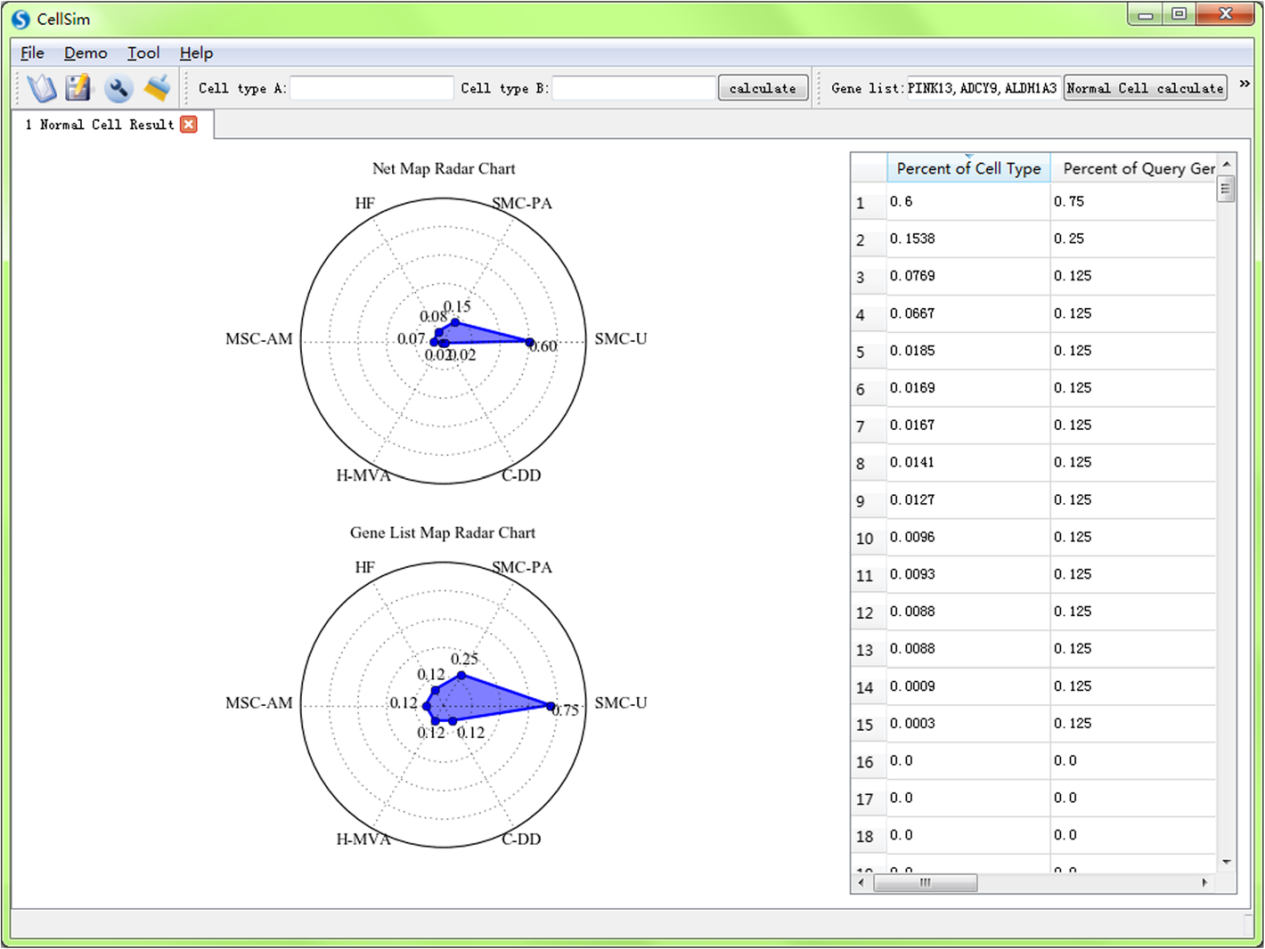
Example using: cell type prediction

We made an example use of cell type prediction (Fig. 8). Specific gene list can be inputted as a file (Fig. 6b) or entered directly from the main screen. In order to get more robust results, we suggest user choose more than 10 genes as input in CellSim for a more accurate prediction result. In order to get an accurate result, the query is divided into two types: normal human cells and cancer cells. The predictions are presented in the main window as individual tabs (Fig. 8). Rader map is made to show the prediction results directly, including the ratio of the sharing genes to cell-specific genes and the ratio of the sharing genes to query genes. These figures can be modified freely by the figure tools in CellSim including title name, axis name, color, transparency and so on. Quantized prediction results are shown as a table on the right. We make a detailed table using the screen the top ten terms (Table 3).

**Table 3:** The top ten predicted cell types of query gene list

Percent of cell type	Percent of query gene list	Cell type
0.6	0.75	smooth muscle cells - uterine
0.1538	0.25	smooth muscle cells - pulmonary artery
0.0769	0.125	heart fetal
0.0667	0.125	mesenchymal stem cells - amniotic membrane
0.0556	0.125	myoblast
0.0323	0.125	renal proximal tubular epithelial cell
0.0244	0.125	fibroblast - lymphatic
0.0185	0.125	heart - mitral valve adult
0.0169	0.125	chondrocyte - de diff
0.0169	0.125	thyroid fetal

## Conclusion

CellSim is a user-friendly and open-source software for the similarity calculation of different cells and the prediction of cell types based on networks which include the structure in Cell Ontology and the cell-specific TF-gene regulation network in FANTOM. This tool will be helpful for the research of cell direct reprogramming and the cellular heterogeneity of cancer cells, especially after the era of human cell atlas researches [32].Through validation of cluster analysis, our computational strategy showed high tidiness and robust in different datasets. CellSim outputs can be downloaded freely, including figures and tables. Integrate other information, including DNA methylation, non-coding RNA regulation and some other source, will be helpful for the cell similarity calculation.

## Abbreviations

ESCs: Embryonic stem cells
IC: Information content
TFs: Transcription factors
